# Trade-off Between Quality, Price, and Profit Orientation in Germany’s Nursing Homes

**DOI:** 10.1007/s12126-015-9227-1

**Published:** 2015-10-17

**Authors:** Max Geraedts, Charlene Harrington, Daniel Schumacher, Rike Kraska

**Affiliations:** Institute for Health Systems Research, School of Medicine, Faculty of Health, Witten/Herdecke University, Alfred-Herrhausen-Strasse 50, 58448 Witten, Germany; Department of Social and Behavioral Sciences, University of California, San Francisco, 3333 California St., Suite 410, San Francisco, CA 94143 USA

**Keywords:** Nursing homes, Germany, Ownership, Profit orientation, Quality of care

## Abstract

International data suggest that for-profit nursing homes tend to provide lower quality than not-for-profit nursing homes. In Germany, the relationships between profit orientation, price and quality of nursing homes have not been investigated. We performed an observational study using secondary data from statutory quality audits of all nursing homes in Germany. The relationships were analyzed bivariately via Mann–Whitney *U*-Test and Kruskal-Wallis Test respectively, followed by a multivariate variance analysis which also covered the interaction effect between quality, price and type of ownership. 41 % of 10,168 German nursing homes were for-profit charging on average about 10 % less than not-for-profit homes. In five out of six quality categories under study, for-profit nursing homes provided lower quality than not-for-profit homes. Quality of care in all quality categories improved with increasing prices per day. However, for four out of six quality categories examined, the quality difference between for-profit and non-profit nursing homes existed independent of the price charged. When selecting a nursing home it is therefore advisable to consider the profit orientation of the institution. German legislation should require that statutory public quality reports contain details on the profit orientation of nursing homes.

## Introduction

Germany, like other industrialised nations, is faced with a steadily increasing risk of need for long-term-care services as a consequence of demographic change. A growing number of people in old age depend on support for activities of daily living (ADL). Most citizens prefer to receive care in their own homes, but towards the end of life many cannot manage without in-patient care in a nursing facility. A growing number of citizens must address the question as to which nursing home is suitable for their individual requirements.

In Germany an obligatory audit for all nursing homes that was introduced in 2009 facilitates this choice; the health insurance funds’ medical service departments evaluate institutional care facilities at least once per year on site, based on a statutory checklist of 82 criteria. Nursing homes are required by law to display audit results on the premises, and statutory nursing insurance funds publish all results via internet.

Long-term-care (LTC) insurance was introduced in Germany in 1995 as a mandatory element of all statutory and private health insurance schemes (Geraedts et al. 2000), so that the entire population is now protected against long-term-care costs. Concurrent with the introduction of LTC insurance, the government started to promote the building and expansion of outpatient and inpatient long-term-care facilities. In this context state funding was available to public non-profit or charitable providers of long-term-care as well as private for-profit providers. As a consequence, the share of for-profit providers of nursing care among outpatient nursing services in Germany increased from 51 to 64 %, and among nursing homes from 35 to 41 % between 1999 and 2013 (Statistisches Bundesamt 2001, 2014).

In a market they share with non-profit providers, for-profit providers of nursing care have to contend with the basic problem of higher capital costs, which may partially be offset by more efficient management, higher prices or diminishing service quality levels. It is generally difficult for users to assess service quality in the case of experience or credence goods such as nursing services. This is why for-profit providers might be induced to deliberately compromise quality in favour of profit maximisation in this sector (Hansmann 1980). Moreover, the most efficient strategy appears to be cost leadership, since other basic competitive strategies such as differentiation and niche strategies are not very promising in a market shared with not-for-profit service providers (Porter 1980). International literature on the correlation between profit orientation of nursing homes and the quality of services provided supports the evidence of these theoretical reflections for the most part. In a meta-analysis, Comondore et al. (2009) concluded that non-profit nursing homes generally provide better care compared to for-profit nursing homes. More recent studies confirm the theoretically expected behaviour on the part of for-profit nursing care providers (Harrington et al. 2012, Harrington et al. 2015). Based on the convincing data available, the US Center for Medicare Advocacy advises consumers to consider profit orientation in the choice of a nursing home, and to select a not-for-profit nursing care facility (Medicare Advocacy 2015).

In view of the above, this study aimed to explore the correlation between profit orientation of German nursing homes, their prices and the quality of care provided.

## Methods

The study was designed as a cross-sectional study based on secondary data. Data sources, operationalisation of variables and analytical procedures are described in the following.

### Nursing Homes

The study was based on the most up-to-date assessments of all German nursing homes in the years 2011 and 2012 respectively from the statutory quality audits performed by the health insurers’ medical service departments. Data were provided by Germany’s largest nursing care insurer, the national sickness fund association AOK (Allgemeine OrtsKrankenkasse), and cover a total of *N* = 10,471 nursing homes offering full inpatient care. Excluded from these data were homes specializing in vigil coma patients (*N* = 36), those with no identifiable type of ownership (*N* = 43), homes with incomplete quality data by more than 50 % (*N* = 2), or with no data on monthly charges for nursing care (*N* = 222). The number of nursing homes remaining for the analysis was *N* = 10,168.

### Profit Orientation

Data on the type of ownership were available for each nursing home under consideration. A distinction was made between private for-profit institutions and public not-for profit and charitable facilities. Because only 5 % of all German nursing homes have public ownership, they were combined with charitable homes in the not-for-profit category for the analysis.

### Prices

Available cost details for each facility cover daily costs for nursing care, room and board. Investment costs which nursing homes charge to each home resident proportionally and on a small scale are not included in the data and therefore do not enter price calculations for each care facility. Moreover, prices per resident depend on the extent of care required. Residents are assigned to one of three care levels; the national distribution of care levels in nursing homes is as follows: care level 1 (lowest) - 38.12 %, care level 2 (medium) – 40.29 %, care level 3 (highest) – 20.45 %. The percentage per nursing home of care recipients assigned to care levels 1 to 3 was not given, so that an equal distribution was assumed, and the daily price per nursing home was calculated as weighted mean of the national distribution.

Average prices charged by nursing homes differ greatly between the 16 federal states in Germany. This is why 5 price categories per federal state were set up with approximately equal numbers of nursing homes for the calculation of correlations between prices and quality of service. As a next step all nursing homes in each of the five price categories were summarized across the nation, and the price was used for analysis as a categorical variable.

### Quality of Care

To assess the quality of care provided in nursing homes the authors used evaluations from the health insurers’ medical service departments collected in unannounced audits in 2011/2012. Audits are based on a checklist of 82 criteria (GKV-Spitzenverband 2008). Eighteen criteria relate to satisfaction surveys among home residents; 38 criteria are determined from a sample of 5–15 residents, depending on the size of the facility; the remaining criteria are assessed per facility. In case of resident-related criteria, the values for individual residents are summed up to form a final value per criterion on a scale from 1 to 5. Facility-related criteria are dichotomous, i.e., registered as either existent or non-existent.

For the purposes of this study, criteria were assigned in terms of content to the following categories: facility structure (4 criteria, e.g., “secured recreational areas outside”, “food and drinks provided in a pleasant environment”), nursing processes (23 criteria, e.g., “required pressure sore prophylaxis is implemented”, “systematic pain assessments conducted”, “biography of residents suffering dementia taken into account and being considered when planning daily activities”), support procedures (16 criteria, e.g., “contact with relatives ensured”, “assistance or information provided to familiarize new residents with the nursing facility”), documentation of nursing services (7 criteria, e.g., “individual risk of falling registered”, “individual risks and resources of residents with incontinence or a bladder catheter assessed”), patient outcomes (2 criteria, “nutritional status appropriate”, “supply of fluids appropriate”) and quality management (5 criteria, e.g., “written instructions available on how to proceed in emergencies”, “nursing facility has a system for managing complaints”). Five criteria were not included in the analysis since over 50 % of pertinent data were not available. Inclusion of two further criteria was not possible since the underlying scale did not permit assignment to suitable content categories. Criteria related to surveyed resident satisfaction were not used either, since these surveys were performed on the basis of a non-validated interview tool and with an insufficient case number and sample size in most cases (Hasseler et al. 2010).

### Statistics

The relationships between profit orientation and prices charged by nursing homes on the one hand, and quality of care on the other were first determined bivariately via Mann–Whitney *U*-Test and Kruskal-Wallis Test respectively, followed by a multivariate variance analysis which also covered the interaction effect between price and type of ownership. Additionally, post hoc tests with Bonferroni-Holm correction for multiple testing were performed to discover the trend of correlations between variables in detail. The statistics software SPSS version 22 was used for all descriptive and analytical evaluations.

## Results

Table 1 shows the absolute and relative frequency of for-profit and non-profit nursing homes in Germany and average daily prices for long-term-care services, room and board. Daily rates charged by the 40 % for-profit homes undercut those charged by non-profit facilities by an average of € 9.17, i.e., more than 10 %.

**Table 1:** Frequency and price per day of for-profit and non-profit nursing homes in Germany

	For-Profit Nursing Homes		Non-Profit Nursing Homes		Total
	N	%	N	%	
Frequency	4179	41.1	5989	58.9	10,168
	Mean	SD*	Mean	SD	Min-Max^#^
Average price per day	71.59 €	9.43 €	80.76 €	13.09 €	35–549 €

*SD standard deviation, *Min*-*Max* ^#^ minimum – maximum price per day

Table 2 lists the average prices charged per day by nursing homes in the five price categories. The large spans result from the pooling of nursing homes from different federal states. The two quintiles 1 and 2 with lowest costs comprise 69 % of for-profit homes but only about 20 % of non-profit facilities.

**Table 2:** Average price per day and number of for-profit and non-profit nursing homes per quintile

	Mean €	Median €	Min €	Max €	N	N for-profit	N non-profit
Quintile 1	67.59	67.79	35.17	82.01	2029	1664	365
Quintile 2	73.28	75.42	58.56	86.06	2033	1216	817
Quintile 3	76.68	78.91	60.46	89.48	2033	738	1295
Quintile 4	80.16	81.80	61.89	93.67	2033	362	1671
Quintile 5	87.19	87.67	65.63	549.06	2040	199	1841
Total	76.98	78.32	35.17	549.06	10,168	4179	5989

Table 3 illustrates the bivariate correlation between profit orientation of nursing homes and the quality of care provided. With the exception of the category “patient outcomes” with only two single criteria, the Mann–Whitney *U*-Test reveals highly significant differences in all other quality categories: non-profit nursing homes consistently provide better quality of care compared to for-profit facilities.

**Table 3:** Relationship between profit orientation and quality of care in nursing homes in Germany (Mann–Whitney *U*-Test)

Quality category (No of criteria)	For-profit	Non-profit	*p*-value
Nursing processes (23)	4.29^a^	4.40^a^	0.000
Documentation (7)	4.49^a^	4.58^a^	0.000
Outcomes (2)	4.93^a^	4.94^a^	0.141
Support services (16)	97.75^b^	98.95^b^	0.000
Quality management (5)	93.20^b^	96.26^b^	0.000
Structures (4)	98.35^b^	99.11^b^	0.000

^a^average value of all nursing homes on a scale from 1 = worst to 5 = best

^b^average value of all nursing homes on a scale from 0 % = worst to 100 % = best

Table 4 shows the bivariate correlation between the five price quintiles and quality categories. The effect that the service quality in German nursing homes increases with increasing prices applies to all categories.

**Table 4:** Relationship between price per day quintiles and quality of care in nursing homes in Germany (Kruskal-Wallis Test)

Quality category (No of criteria)	Quintile 1	Quintile 2	Quintile 3	Quintile 4	Quintile 5	*p*-value
Nursing processes (23)	4.23^a^	4.30^a^	4.36^a^	4.41^a^	4.47^a^	0.000
Documentation (7)	4.43^a^	4.50^a^	4.55^a^	4.58^a^	4.64^a^	0.000
Outcomes (2)	4.92^a^	4.93^a^	4.94^a^	4.94^a^	4.95^a^	0.009
Support services (16)	97.19^b^	98.40^b^	98.79^b^	98.84^b^	99.08^b^	0.000
Quality management (5)	91.82^b^	94.90^b^	95.38^b^	96.02^b^	96.90^b^	0.000
Structures (4)	97.86^b^	98.82^b^	98.93^b^	99.07^b^	99.29^b^	0.000

^a^average value of all nursing homes on a scale from 1 = worst to 5 = best

^b^average value of all nursing homes on a scale from 0 % = worst to 100 % = best

Table 5 shows the results of the multi-variate variance analysis. The figure below additionally illustrates correlations between prices charged by nursing homes, profit orientation and the six quality categories examined (see figure). Irrespective of profit orientation, the quality of care in all quality categories improves with increasing prices per day, the same as in the bivariate analysis. However, profit orientation cannot be considered as independent of the price in all quality categories. In the categories of nursing processes, support services, quality management and structures, for-profit homes perform worse than non-profit institutions, independent of the price charged. No correlations were found, however, between documentation quality and outcomes on the one hand and profit orientation of the facilities on the other if the price charged by the nursing homes is taken into consideration (see Table 5).

**Table 5:** *P*-values and variance explained of the multivariate relationship between price per day quintiles, profit orientation and quality of care and their interactions in nursing homes in Germany

Quality category (No of criteria)	Price per day	Profit orientation	Price per day*profit orientation	R^2^
Nursing processes (23)	0.000	0.028	*0.199*	0.022
Documentation (7)	0.000	*0.419*	*0.571*	0.018
Outcomes (2)	0.001	*0.373*	*0.894*	0.003
Support services (16)	0.000	0.000	0.013	0.026
Quality management (5)	0.000	0.000	0.000	0.028
Structures (4)	0.005	0.004	0.011	0.009

Significant interactions between price and type of ownership were found for the criteria support services, quality management and structures. The three graphs on the right side of the figure below illustrate that for-profit nursing homes effect stronger quality improvement between the 1st and 2nd quintiles compared to non-profit homes (see figure). But for-profit homes achieve a higher quality level than non-profit homes in only one quintile for these categories.

Across all six quality categories, the level of quality in for-profit nursing homes is lower than in non-profit homes in 23 out of 30 comparable price quintiles. Additional post hoc tests with Bonferroni-Holm correction confirm the graphic impression that there are significant differences in relation to profit orientation mainly in the lower price quintiles, whereas the upper price quintile does no longer reveal any statistically significant differences. It also appears that a good part of significant improvement happens up to the third price quintile across all quality categories, and the area beyond shows only consistently significant quality improvement for nursing processes.

**Fig. 1:**
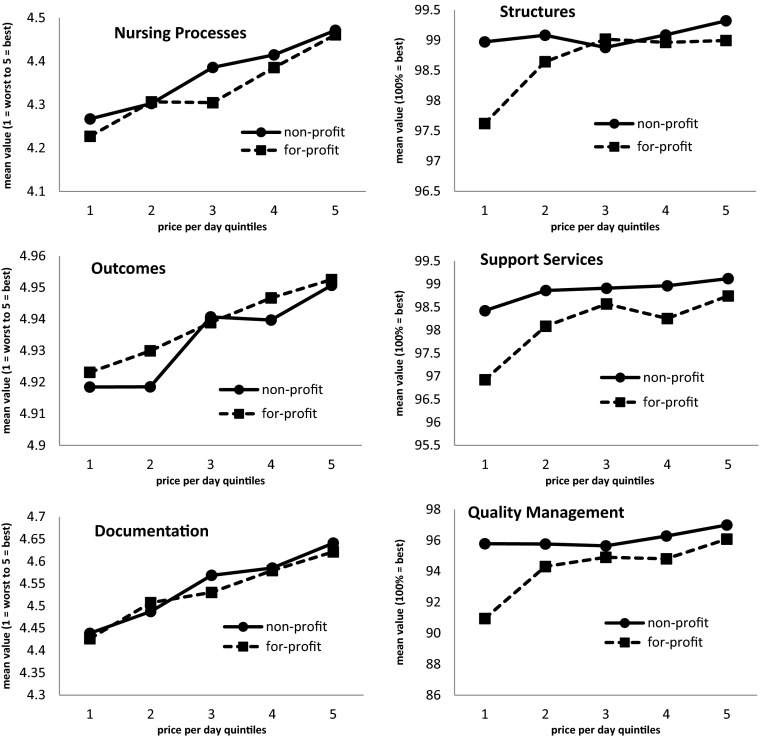
Quality of care in six categories in relation to price per day and profit orientation of nursing homes in Germany (*N* = 10,168) (note that Y-axis and X-axis scaling varies)

Based on a summary of results shown in Tables 3, 4 and 5 and the figure it must be acknowledged that all identified differences are at a low level and that nursing homes in general have received very good ratings. Therefore the multivariate model only explains approximately 3 % in the variance of quality differences between for-profit and non-profit nursing homes, and between nursing homes of different price quintiles Fig. 1.

## Discussion

All in all, for-profit nursing homes in Germany offer a lower service quality compared to non-profit nursing homes. One explanation for the differences is that for-profit homes charge lower prices on average and that the quality of nursing services is related to the price to a highly significant degree. However, for four out of six quality categories examined, the quality difference between for-profit and non-profit nursing homes exists independent of the price charged. Therefore German for-profit nursing homes mainly appear to pursue the strategy of cost leadership described by M. Porter (1980) and accept the quality concessions involved.

Study results from Germany confirm findings described in the international literature which also identified a lower level of quality in for-profit nursing homes (Comondore et al. 2009, Harrington et al. 2012, Harrington et al. 2015). Also confirmed are results from a German study which found a correlation between prices charged by nursing homes and the quality of services provided when examining a smaller sample size of nursing homes (Mennicken 2013). A new finding from the present study is that quality differences between German nursing homes exist independent of the price. In the lower price bracket in particular, the average quality of for-profit nursing homes was found to be considerably below the quality in non-profit nursing homes of the same segment, whereas almost no differences between nursing homes were found in the upmost price segment in relation to profit orientation.

An analysis of the reported interaction between prices charged by nursing homes and their profit orientation suggests the following interpretation: specifically in the case of for-profit homes, it is worth selecting a nursing home from a higher price category to hope that the level of quality will be comparable to the care in non-profit homes. In the case of non-profit facilities, however, the price charged plays a minor role only in terms of quality. The lowest price category has many nursing homes that offer quality of the highest level.

### Limitations

Several limitations must be considered in the interpretation of study results. First, the design of a cross-sectional study does not permit to establish a causal relation. It is true that the quoted economic theories of Porter (1980) and Hansmann (1980) suggest the existence of such a relation. But the study design only permits to ascertain that the average for-profit nursing home in Germany, the same as elsewhere, offers services of a lower quality compared to non-profit institutions. The question remains open as to whether profit orientation is responsible for this fact.

An important limitation is inherent in the system of assessing nursing home quality in Germany. The majority of assessment criteria have a low discriminative potential, so that almost all nursing homes receive good to very good ratings. Moreover, outcome quality is hardly ascertained. Because the outcome measures did not show differences in facilities, perhaps the outcome measures should be reexamined for reliability and validity to determine whether they can distinguish quality among facilities. These points of criticism have been raised in Germany for years (Hasseler et al. 2010). But a partial revision of the catalogue of criteria has not included a comprehensive reform of assessment procedures which was repeatedly postponed by health politicians in the past. Pending this revision, researchers depend on the data available. Thanks to the fact that a full assessment of all nursing homes and an unbiased large-scale sample was available for analysis, it was nevertheless possible to statistically secure quality differences between nursing homes associated with profit orientation.

Another limiting aspect was the impossibility to consider the distribution of nursing requirements among residents of each home in the analysis since no pertinent data were available. All nursing homes were therefore assumed to have an individual distribution of care levels corresponding to the national average, i.e., approximately 40 % of residents assigned to care levels one and two, and 20 % to care level three. It is unlikely that this distribution differs between for-profit and non-profit homes in Germany in such a way that for-profit nursing homes have more residents in need of intensive nursing care. This is why it may be assumed that differing compositions of residents in terms of care levels do not explain quality differences in relation to profit orientation.

A methodological limitation is the fact that requirements for the multivariate variance analysis employed in the study were not met. On the one hand, there was no multivariate normal distribution; on the other, it was unclear whether the variance-covariance matrices were sufficiently homogeneous. It may be stated that there was a high robustness in terms of the alpha error in view of the large sample available. Moreover, additional non-parametric tests yielded the same results so that the MANOVA results may be interpreted in the presented form.

## Conclusions

For-profit nursing homes in Germany, as shown in other countries, offer a lower level of quality compared to non-profit homes. The price charged by nursing facilities is related to quality: higher quality can be expected for higher prices, which applies mainly to for-profit facilities. When selecting a nursing home it is therefore advisable to consider the profit orientation of the institution. German legislation should require that statutory public quality reports contain details on the profit orientation of nursing homes, which is reported on the U.S. government’s nursing home quality report card (CMS 2015).
